# Sex-specific familial aggregation of cancers in Finland

**DOI:** 10.1038/s41598-022-19039-1

**Published:** 2022-09-06

**Authors:** Lauri J. Sipilä, Karri Seppä, Mervi Aavikko, Janne Ravantti, Sanna Heikkinen, Lauri A. Aaltonen, Janne Pitkäniemi

**Affiliations:** 1 Department of Medical and Clinical Genetics, Biomedicum Helsinki, University of Helsinki, Haartmaninkatu 8, PO Box 63, 00014 Helsinki, Finland; 2 Applied Tumor Genomics Research Program, Research Programs Unit, Biomedicum Helsinki, University of Helsinki, Haartmaninkatu 8, PO Box 63, 00014 Helsinki, Finland; 3 Finnish Cancer Registry, Unioninkatu 22, 00130 Helsinki, Finland; 4 Institute for Molecular Medicine Finland (FIMM), HiLIFE, University of Helsinki, Helsinki, Finland; 5 Molecular and Integrative Biosciences Research Programme, Faculty of Biological and Environmental Sciences, University of Helsinki, 00014 Helsinki, Finland; 6 Health Sciences Unit, Faculty of Social Sciences (Health Sciences), Tampere University, Tampere, Finland; 7 Department of Public Health, Faculty of Medicine, University of Helsinki, Helsinki, Finland; 8 Institute for Statistical and Epidemiological Cancer Research, Finnish Cancer Registry, Unioninkatu 22, 00130 Helsinki, Finland

**Keywords:** Cancer epidemiology, Risk factors

## Abstract

Despite the fact that the effect of sex on the occurrence of cancers has been studied extensively, it remains unclear whether sex modifies familial aggregation of cancers. We explored sex-specific familial aggregation of cancers in a large population-based historical cohort study. We combined cancer and population registry data, inferring familial relationships from birth municipality-surname-sex (MNS) combinations. Our data consisted of 391,529 incident primary cancers in 377,210 individuals with 319,872 different MNS combinations. Cumulative sex-specific numbers of cancers were compared to expected cumulative incidence. Familial cancer risks were similar between the sexes in our population-wide analysis. Families with concordant cancer in both sexes exhibited similar sex-specific cancer risks. However, some families had exceptionally high sex-specific cumulative cancer incidence. We identified six families with exceptionally strong aggregation in males: three families with thyroid cancer (ratio between observed and expected incidence 184.6; 95% credible interval (95% CI) 33.1–1012.7, 173.4 (95% CI 65.4–374.3), and 161.4 (95% CI 29.6–785.7), one with stomach (ratio 14.4 (95% CI 6.9–37.2)), colon (ratio 15.5 (95% CI 5.7–56.3)) cancers and one with chronic lymphocytic leukaemia (ratio 33.5 (95% CI 17.2–207.6)). Our results imply that familial aggregation of cancers shows no sex-specific preference. However, the atypical sex-specific aggregation of stomach cancer, colon cancer, thyroid cancer and chronic lymphocytic leukaemia in certain families is difficult to fully explain with present knowledge of possible causes, and could yield useful knowledge if explored further.

## Introduction

Global disparities in the incidence of cancers between males and females are well documented^1^. Incidence differences of varying magnitude between the sexes are observed in almost all cancers: yearly incidence rates are greater in men in almost all non-sex-specific malignancies, except thyroid cancer^2,3^. This phenomenon has classically been explained by biological differences, in addition to unequal exposure to environmental, lifestyle and occupational factors. For example, in Sweden, peaks in lung cancer incidence over time have followed the divergent prevalence peaks of smoking in males and females^4^. The contribution of biological mechanisms is, for example, supported by patterns of sex differences in childhood cancers^5^. Sex-dependent modification of environmental effects also occurs, as for example the incidence rate of hepatocellular carcinoma is different in men and women with chronic hepatitis B infection, but not with hepatitis C infection^6^.

Familial aggregation of cancer can be caused by both genetic and shared environmental factors^7^, as well as stochastic phenomena. The offspring and siblings of cancer patients are at a significantly increased risk of cancer^8,9^. Familial aggregation can also be modified by parental lifestyles, such as physical inactivity, being passed onward to children^10^.

Sex does not appear to greatly modify the risk of familial cancer, as parent–offspring pairs of concordant cancer patients in Sweden exhibited abnormal sex-specific aggregation of only thyroid adenocarcinoma in male offspring of the proband^11^. Singular studies of familial cancers have observed sex-related aggregation effects^12,13^.

While sex disparities of cancer result from a complex interplay of different factors^14^, in some cases cancer may result rather directly due to predisposing genetic variants in the sex chromosomes, as a few X-linked cancer syndromes have been described^15^. Mutations in sex chromosomes have been implicated to have numerous roles in cancer^16^. Thus, it seems plausible that germline variation in sex chromosomes affects lifetime cancer risk as well. However, associations of sex and lifetime risk of familial cancer has been studied rarely. Thus, additional studies are warranted; detecting associations in an epidemiological setting would allow the formulation of additional research hypotheses, which in turn might reveal novel information about cancer biology and may be of importance in monitoring families with cancer.

We studied population-based sex-specific familial aggregation of cancers, inferring familial relationships indicated by shared surnames at birth and birth municipalities of cancer patients. Our model is an extension of the model introduced by Kaasinen et al.^17^ to include sex-specific effects. Finns are a population isolate with a unique history of bottleneck events, and subsequent genetic drift. This, in parallel with the whole-population setting of our study, provides a unique perspective on this seldom studied subject. There appears to be no sex-specific difference in aggregation population-wide, but individual families may be affected by rare phenomena as we observed notable sex-specific cancer clusters.

## Methods

We explored familial sex-specific aggregation of cancers in a population-based historical cohort setting. We used all diagnosed primary cancers reported in the Finnish Cancer Registry (FCR) starting from 1953 up until the end of 2016. Cancers were classified on the basis of topography, morphology and behaviour codes based on ICD-O-3, and grouped to ICD-10-equivalent groups according to official FCR classification^18,19^. From this data we filtered non-sex-specific cancer types that are recorded and reported in FCR’s yearly statistics. Breast cancer, which can also occur in males, was left out of the analysis due to the very large observation differential. Of the hematologic malignancies, we included major ICD-10 cancer types that had a long and reliable time series in the registry, dating back to at least the 1960s. Non-hodgkin lymphomas were excluded from the analysis as these are separated in FCR’s yearly statistics, and many of the malignancies comprising this group are individually too rare for reliable inference. The Digital and Population Data Service Agency of Finland collects and stores data in the Finnish Population Information System (PIS) of people who are born in or immigrate to Finland, until their emigration or death. From PIS we obtained the number of people born in each municipality, with each surname, of each sex and in each year, totaling 8,256,557 people (DVV permit 617/410/07). People were tabulated by municipality, name, and sex, creating 1 774 080 municipality-name-sex (MNS) combinations. The combination of the same birth municipality and surname at birth was the basis for inference of familiality^17,20^. A minimum of two people for each combination was required to reduce computational burden and stabilise the statistical inference, reducing the number to 609,406 MNS combinations.

We linked the personal identity codes of cancer patients (FCR data, permit SSY-2011-0002) to PIS data to retrieve their surnames at birth and birth municipalities (DVV permit VRK/41405/2017-2). We limited cancer patients to those born between 1870 and 2010 to reduce random variation from very small birth cohorts. We restricted the age of onset of cancers to below 80 years of age, as cancer diagnoses tend to be less reliable in the very elderly. Only primary cancer diagnoses were considered, once for each cancer type per individual. Twenty-eight study subjects without information on sex were removed. Thus, our data consisted of 391,529 cancer diagnoses in 377,210 individuals (Table 1), in 319,872 MNS combinations.

**Table 1:** Number of cancers, MNS combinations and average number of cancers per MNS in the study population, by cancer site, between 1956 and 2016.

Site	ICD-10	Male			Female			Cancers total	% Cancers by sex (male/female)
		Cancers	MNS^a^ combinations	Average cases per MNS^a^	Cancers	MNS^a^ combinations	Average cases per MNS^a^		
Lip	C00	5090	4702	1.083	919	915	1.004	6009	85/15
Pharynx	C01, C09–14	2749	2660	1.033	1041	1029	1.012	3790	73/27
Tongue	C02	1388	1366	1.016	1134	1115	1.017	2522	55/45
Salivary glands	C07–08	934	920	1.015	944	936	1.009	1878	50/50
Oesophagus	C15	4688	4465	1.05	2708	2623	1.032	7396	63/37
Stomach	C16	18,871	16,232	1.163	12,787	11,470	1.115	31,658	60/40
Small intestine	C17	1425	1406	1.014	1145	1136	1.008	2570	55/45
Colon	C18	17,454	15,237	1.146	19,082	16,708	1.142	36,536	48/52
Rectum and rectosigmoid	C19–20	14,349	12,810	1.12	11,179	10,209	1.095	25,528	56/44
Anus	C21	319	318	1.003	511	509	1.004	830	38/62
Liver	C22	5652	5335	1.059	3067	2970	1.033	8719	65/35
Gallbladder and bile ducts	C23–24	2436	2371	1.027	4686	4486	1.045	7122	34/66
Pancreas	C25	12,430	11,167	1.113	11,142	10,150	1.098	23,572	53/47
Nose and sinuses	C30–31	813	806	1.009	576	572	1.007	1389	59/41
Larynx and epiglottis	C32	5255	5004	1.05	491	489	1.004	5746	91/9
Lung and trachea	C33–34	67,018	45,345	1.478	15,591	13,714	1.137	82,609	81/19
Melanoma of the skin	C43	11,743	10,597	1.108	11,319	10,331	1.096	23,062	51/49
Kidney	C64	12,802	11,529	1.11	9245	8521	1.085	22,047	58/42
Bladder and urinary tract	C65–68, D0.90–1, D41.1–9	20,131	17,271	1.166	5796	5519	1.05	25,927	78/22
Brain, meninges, and central nervous system	C70–72, D32–33, D42–43	11,817	10,822	1.092	15,715	14,065	1.117	27,532	43/57
Thyroid gland	C73	2808	2728	1.029	9468	8705	1.088	12,276	23/77
Hodgkin lymphoma	C81	3167	3073	1.031	2331	2281	1.022	5498	58/42
Myeloma and other plasma cell tumours	C90	4434	4262	1.04	4485	4303	1.042	8919	50/50
Acute lymphoblastic leukaemia/lymphoma	C91.0	1471	1445	1.018	1198	1183	1.013	2669	55/45
Chronic lymphocytic leukaemia	C91.1	5275	4961	1.063	3636	3505	1.037	8911	59/41
Acute myeloid leukaemia	C92.0	2507	2455	1.021	2372	2316	1.024	4879	51/49
Chronic myeloid leukaemia	C92.1	1055	1038	1.016	880	876	1.005	1935	55/45

^a^Municipality-name-sex combination.

The study protocol, its use of human data, and methodology have been evaluated and approved by the Finnish Institute for Health and Welfare (THL permit 151/5.05.00/2017) and the Ethics Committee of the Hospital district of Helsinki and Uusimaa (HUS/2509/2016). All work involving patient data was carried out in accordance with the Declaration of Helsinki.

Informed consent from study participants was unnecessary as the study utilised administrative registry data not based on consent (based on the Act on the Finnish Institute for Health and Welfare 668/2008 (cancer information), Act on the Population Information System 661/2009 (other than health information) and the Act on the Secondary Use of Health and Social Data (552/2019)).

The observed number of cancers in each MNS combination was compared with an expected number, which was obtained by applying the standard cancer incidence in Finland to the MNS combination by birth cohort. The ratio between the observed and the expected number until the end of follow-up (end of 2016) is called standardised cumulative incidence ratio (SCIR) as it follows the approach of standardised incidence ratio (SIR)^21^, with the exception that cumulative incidences are utilised instead of age-specific rates. SCIR was used to quantify cancer risk in each MNS combination compared to the average risk in the population.

SCIRs were modelled using a hierarchical Bayesian Poisson regression. Based on the distribution of SCIRs in site-specific MNS combinations, we simulated two risk coefficients for each combination. The regression model was fitted separately to the data of each site and sex using Markov chain Monte Carlo simulation. Bayesian hierarchical modelling was used as a statistical smoothing method to manage the problem of multiple comparisons due to the large number of combinations of family name and municipality. The computation of all MNS combinations is prohibitively cumbersome due to their large number, and because the aim of this study was to differentiate inferred families by their cancer risk, we excluded from each analysis all MNS combinations with no cancers. The model, prior distributions, details of the simulation, and a directed acyclic graph of the model are available in Online Resource 1.

For each site and sex, we estimated the proportion of cancers in the high versus low risk coefficient groups (risk coefficient group proportion), SCIR averages both overall and in the higher coefficient group, and the ratio of these SCIR averages (SCIR ratio). We compared derived proportions of groups between sites and sexes, based on the assumption that SCIR distribution shape, and thus risk coefficient group proportion, was insensitive to the scale of SCIRs within each analysis. We estimated male–female ratios of the risk coefficient group proportion and the SCIR ratio.

In addition, we calculated cancer-specific posterior probabilities of the risk coefficient group proportion and normalised SCIR estimates in each MNS combination. We compared the amount of MNS combinations with higher risk coefficient posterior probability over 0.9 between males and females. We examined the full detailed cancer histories for MNS combinations with higher risk coefficient posterior probability over 0.95. For SCIRs we chose to use the median as a summary statistic as some SCIR posterior distributions were skewed, in which cases the arithmetic mean was shifted towards the tail of the distribution and the median was more robust. Normalised SCIRs were derived from the posterior medians of MNS combination SCIRs by subtracting each MNS combination’s SCIR median from its respective cancer-sex analysis set’s median SCIR, and dividing it by the standard deviation. Normalised SCIR thus measures an MNS combination’s divergence from the site-and-sex SCIR median as the number of standard deviations from the median, enabling the detection of atypical SCIRs. A subset of MN combinations presenting concordant cancer in both sexes was extracted from the data to study intra-family effects, yielding 20,925 such combinations with a total of 61,855 individuals.

Data processing was done using R version 3.5.1 utilising the package data.table version 1.12.2. Figures were generated using R package ggplot2 version 3.2.1^22^.

## Results

Cancer sites for which more than 55% of all diagnosed individuals were female included the gallbladder and bile ducts, anus, thyroid gland, as well as brain, meninges, and central nervous system (CNS, Table 1). Similar numbers of cancer in both sexes were observed in salivary gland cancer, colon cancer, melanoma of the skin, myeloma and other plasma cell tumours, and acute myeloid leukaemia (AML). The remaining 18 sites had an excess of diagnoses in males. Males had on average a much higher excess number of familial cancers in the lung and trachea (1.48 cancers per MNS vs. 1.14 in females). The thyroid gland was the only site with a noticeable excess of familial female cancers (1.09 cancers per MNS vs. 1.03 in males).

Male–female ratios of the risk coefficient group proportions and SCIR ratios are shown in Table 2, with complete aggregate results available in Online Resource 2. No differential familial aggregation between sexes was observed. Male–female ratio of SCIR varied from 0.87 (95% credible interval (95% CI) 0.35–1.73) in nose and sinuses to 1.04 (95% CI 0.41–2.19) in chronic myeloid leukaemia. The most deviated male/female ratio of risk coefficient group proportion with reasonable variation was in the lung and trachea (0.83 (95% CI 0.61–1.12)), with a male–female SCIR ratio of 0.95 (95% CI 0.87–1.04). The risk coefficient group proportion of male lung and trachea cancers was 0.53 and for females 0.64 (Online Resource 2).

**Table 2:** Male–female ratios of risk coefficient group proportions and standardised cumulative incidence ratios by cancer site from 1956 to 2016.

Site	Risk coefficient group proportion^a^, male/female	SCIR ratio^b^, male/female
Lip	1.15 (0.61–124.20)	0.90 (0.37–1.21)
Pharynx	1.00 (0.72–38.78)	0.98 (0.43–1.19)
Tongue	1.05 (0.03–101.42)	0.98 (0.37–2.16)
Salivary glands	0.97 (0.02–81.93)	1.00 (0.35–2.46)
Oesophagus	1.05 (0.75–1.60)	0.97 (0.80–1.16)
Stomach	1.07 (0.85–1.40)	0.95 (0.85–1.05)
Small intestine	1.09 (0.01–187.42)	0.95 (0.31–2.55)
Colon	1.07 (0.86–1.36)	0.97 (0.89–1.06)
Rectum, rectosigmoid	1.00 (0.78–1.32)	0.99 (0.88–1.10)
Anus	0.94 (0.03–105.87)	1.04 (0.35–2.61)
Liver	0.95 (0.70–1.31)	1.02 (0.86–1.19)
Gallbladder, bile ducts	1.00 (0.69–1.38)	1.01 (0.86–1.21)
Pancreas	1.04 (0.80–1.34)	0.97 (0.87–1.09)
Nose, sinuses	1.33 (0.23–74.79)	0.87 (0.35–1.73)
Larynx, epiglottis	1.06 (0.69–46.48)	0.93 (0.35–1.19)
Lung, trachea	0.83 (0.61–1.12)	0.95 (0.87–1.04)
Melanoma of the skin	1.00 (0.81–1.24)	1.00 (0.90–1.10)
Kidney	0.99 (0.77–1.28)	0.99 (0.88–1.11)
Bladder and urinary tract	0.91 (0.70–1.21)	0.99 (0.88–1.11)
Brain, meninges and central nervous system	1.06 (0.84–1.32)	0.98 (0.89–1.09)
Thyroid gland	1.06 (0.76–1.37)	0.99 (0.86–1.19)
Hodgkin lymphoma	0.98 (0.75–1.29)	1.01 (0.86–1.18)
Myeloma and other plasma cell tumours	1.02 (0.76–1.40)	0.99 (0.85–1.15)
Acute lymphoblastic leukaemia/lymphoma	0.97 (0.66–1.35)	1.01 (0.83–1.26)
Chronic lymphocytic leukaemia	0.98 (0.74–1.30)	1.00 (0.86–1.15)
Acute myeloid leukaemia	1.01 (0.67–1.53)	0.99 (0.79–1.24)
Chronic myeloid leukaemia	0.93 (0.04–37.03)	1.04 (0.41–2.19)

Male–female ratios of risk coefficient group proportions and standardised cumulative incidence ratios, with 95% posterior credible intervals given in parentheses, by cancer site.

^a^Proportion of municipality-name-sex combinations for which a higher risk coefficient was used during a sampling step.

^b^Standardised cumulative incidence ratio in higher risk coefficient municipality-name-sex combinations over standardised cumulative incidence ratio in all municipality-name-sex combinations.

**Figure 1:**
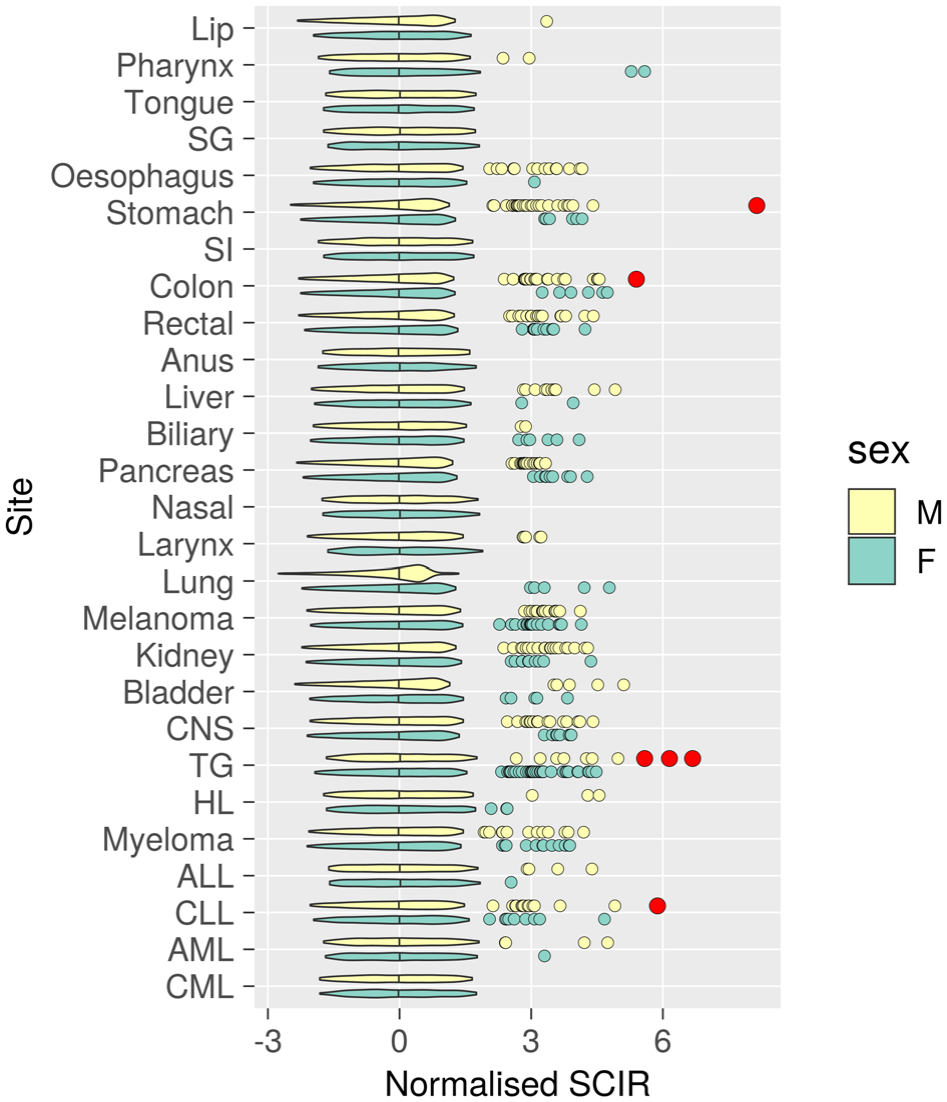
Normalised standardised cumulative incidence ratio (SCIR) estimates of municipality-name-sex combinations. Violin plots for central 95% of normalised SCIRs. MNS combinations allocated to higher risk coefficient group with probability greater than 90% are indicated by dots, with red colour and larger dot size used to emphasise most extreme probabilities (greater than 95%). Sites presented in the same order as in Tables 1 and 2, with abbreviated names. *SG* salivary glands, *SI* small intestine, rectal rectum and rectosigmoid, *biliary* gallbladder and bile ducts, *nasal* nose and sinuses, larynx larynx and epiglottis, *lung* lung and trachea, *melanoma* melanoma of the skin, *urinary* bladder and urinary tract, *CNS* brain, meninges, and central nervous system, *TG* thyroid gland, *HL* Hodgkin lymphoma, *myeloma* myeloma and other plasma cell tumours, *ALL* acute lymphoblastic leukaemia/lymphoma, *CLL* chronic lymphocytic leukaemia, *AML* acute myeloid leukaemia, *CML* chronic myeloid leukaemia.

Next we attempted to detect any atypically aggregating families for all cancer sites by normalising the posterior SCIR medians by each site-and-sex grouping (Fig. 1). In most studied cancers, posterior SCIR median distributions were very similar between the sexes. Male MNS combinations tended to have higher cancer risks. Males had more high-risk MNS combinations in the stomach (21 clusters more than females), colon (16), oesophagus (13), pancreas (11), CNS (10), kidney (10), liver (8), rectum and rectosigmoid (6), CLL (5), larynx and epiglottis (5), AML (3), acute lymphoblastic leukaemia/lymphoma (ALL, 3), lip (1), and myeloma and other plasma cell tumours (1). Females had more high-risk MNS combinations in the thyroid gland (25), lung and trachea (5), melanoma of the skin (4), and gallbladder and bile ducts (4). Posterior probabilities of higher risk coefficient > 0.95 were identified only in male MNS combinations with clustered observations. These were in thyroid cancer (three clusters, with (1) two medullary carcinomas; SCIR 184.6 (95% CI 33.1–1012.7), normalised SCIR 6.68, (2) two papillary carcinomas; SCIR 161.4 (95% CI 29.6–785.7), normalised SCIR 5.6, (3) four medullary carcinomas, one follicular carcinoma, and one epithelial carcinoma; SCIR 173.4 (95% CI 65.4–374.3), normalised SCIR 6.15), colon cancer (one cluster with three adenocarcinomas; SCIR 15.5 (95% CI 5.7–56.3), normalised SCIR 5.40), CLL (one cluster of three individuals; SCIR 33.5 (95% CI 17.2–207.6), normalised SCIR 5.88), and stomach cancer (one cluster, with three adenocarcinomas, two diffuse type adenocarcinomas, and one carcinoid tumour; SCIR 14.4 (95% CI 6.9–37.2), normalised SCIR 8.14). These clusters exhibited no concordant cancer in women, except the six-patient thyroid cancer cluster which included a thyroid cancer observation in one woman. The stomach cancer cluster also co-occurred with two female lung cancers and one Hodgkin lymphoma.

**Figure 2:**
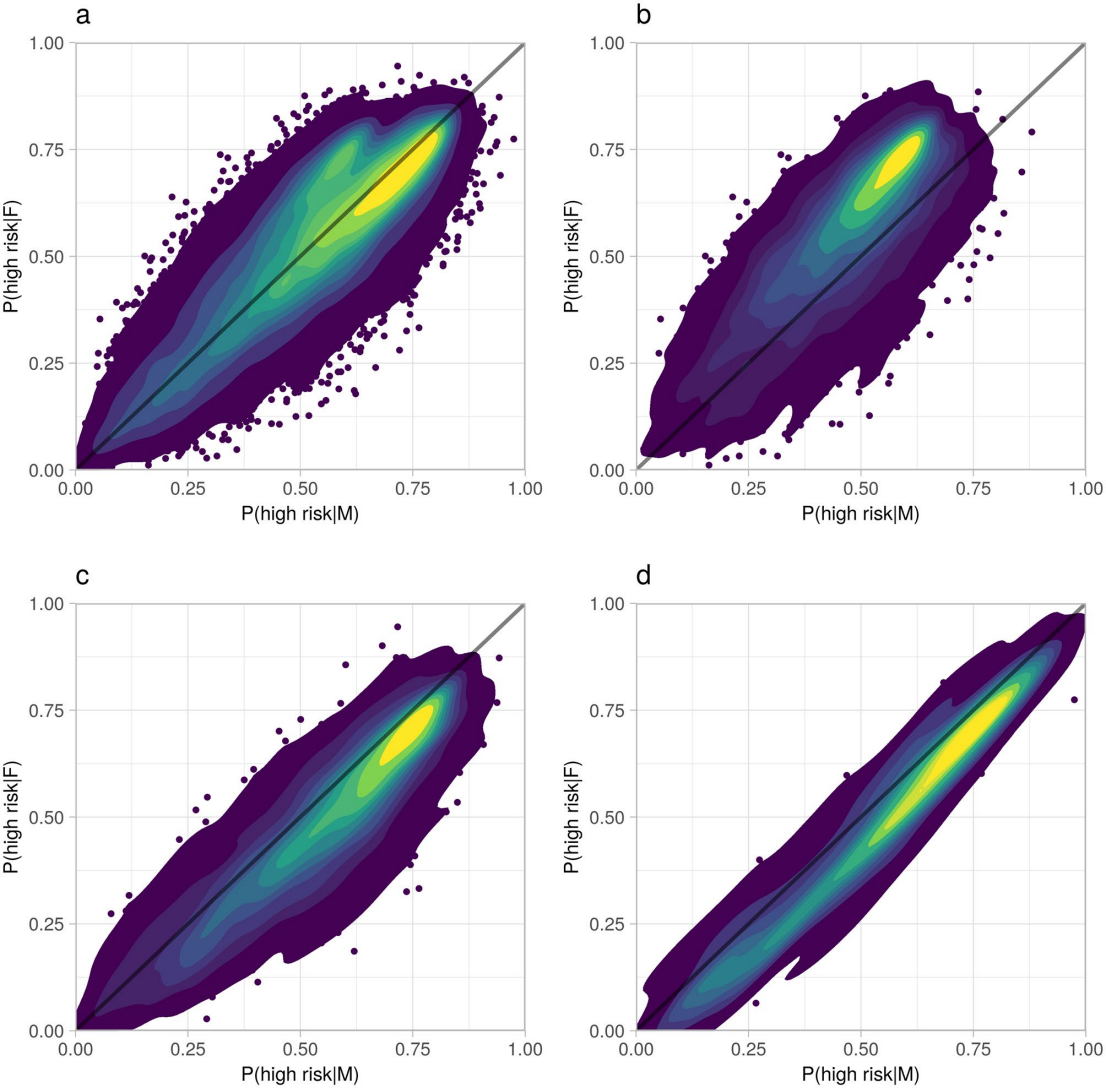
Higher risk coefficient posterior probability (high risk) in males versus in females, in inferred families with concordant cancer. (**a**) All sites, (**b**) lung and trachea, (**c**) colon, (**d**) thyroid gland. Contours start from ~ 99% of data points encompassed, followed by 90%, after which decrements are by each 10%. Observations remaining outside the largest contour visualised as points. Diagonal line drawn as a visual reference for equal male and female risk coefficient posterior probability.

As higher risk coefficient assignment with P > 0.95 was only observed in males, we examined whether sex affected risk coefficient assignment by studying MN combinations with concordant cancer in males and females. Males and females are likely to be categorised similarly in each MN combination. This is indicated in the contour plot in Fig. 2a, in which male and female higher risk coefficient posterior probabilities are plotted for all sites combined. Lung and trachea cancers are numerous enough to show up in the figure as their own separate cluster, which has been isolated in Fig. 2b. In the central 10% of MN combinations with concordant lung cancer in the contour plot, females are ~ 0.15 more likely to be assigned a higher risk coefficient. In colon cancer (Fig. 2c) the contours are skewed towards a higher probability in males, implying a family-level effect similar to the whole-population aggregation result of an elevated male–female ratio of colon cancer risk coefficient group proportion, shown in Table 1. A visualisation of thyroid gland clusters is shown in Fig. 2d to emphasise the sex difference in the cluster with six male observations and one female observation.

## Discussion

We studied sex-specific familial aggregation of cancer with nationwide population-based registry data. No population-wide differential of familial aggregation between males and females was detected in any of the studied cancers. Singular cancer clusters of inferred families emerged, with the largest cancer risk increases observed in male MNS combinations with thyroid cancer, colon cancer, CLL, and stomach cancer. Risk coefficient assignment probabilities of MN combinations with concordant cancer follow a correlated pattern between males and females in our simulations, indicating only minor or rare sex-dependent modification of familial risk.

In this study, we inferred familial relationships from birth surname and birth municipality combinations utilising information extracted from a population registry, an approach which has been previously applied with success in Finnish cancer studies^17,20^. This has allowed us to use all cancers in Finland in the current analysis, with the large number of data points available improving statistical power to detect MNS combinations with high cancer risk. Our statistical modelling accounts for complex random variation between clusters, and decreases false positive findings by utilising Bayesian shrinkage. The high quality of cancer registration for decades and the quality of the population registry decrease biases and variation from these data^23,24^.

As we lack both historical data of population levels in each municipality and the death or emigration years of individuals not present in the FCR, we are unable to calculate person-years at risk in each MNS. This prevents the calculation of expected number of cases by age group and subsequently SIRs, and thus we use SCIR as our measure. Our model estimates SCIR for a cancer only in MNS combinations with observations, while expected cancer rates are calculated based on the whole population prior to inference. This biases the model offset, which in turn overestimates risk with a varying degree and makes direct comparison of different sex-and-site analyses unfeasible. Thus, we mainly use our sex-and-site-specific risk metrics as intermediate results when describing effects between sexes and inferred families. The purpose of our dichotomous risk categorisation in simulations was to manage the low number of cancers in each MNS combination, due to which the SCIR estimates were unstable. This limitation was counteracted by the large number and high variability of families and family sizes, with which we were able to identify the distributions of SCIRs in MNS combinations for each cancer site.

The method of inferring familial relationships by surname at birth has two challenges. First, common surnames can be shared by multiple individuals with no particular familial relation, leading to inevitable misclassification of some unrelated individuals as being related. The range of surnames in use is large, however. For example, according to DVV open data, the 50 most common surnames in 2020 accounted for approximately 6.5% of the population, and the 10 most common for 1.9%. Familial misclassification is considered to be non-differential, as healthy individuals and persons with cancer are misclassified similarly. Previous studies using non-sex-specific MN combinations inferred from surname and birth municipality^17,20^ have validated their findings with genealogy, with the majority of inferred families in observed high-risk clusters consisting of first-degree relatives.

The second challenge is that some MNS combinations and cancer families are misclassified as affected children might not have the same surname at birth as the affected same-sex parent. Finnish women have customarily taken their husband’s surname upon marriage, and children born in the marriage typically receive their father’s surname at birth. Similarly, male MNS combinations may be misclassified if a male offspring is born out of wedlock, as they may receive the mother’s surname. The magnitude of this effect is difficult to assess exhaustively. Changing surname upon marriage was mandatory through the 1930s to 1980s, but before this time the custom has varied in adoption over time, by region, and by socioeconomic group^25^. Children born out of wedlock have received their mother’s surname by default. Children born out of wedlock numbered 7–9% from the late nineteenth century until the Soviet-Finnish wars, after which the rate reduced to around 5% until the 1970s^26^. Since then the rate has steadily increased to a contemporary rate of 40–45% according to data from Statistics Finland. Both female and male MNS combinations had a median size of 12.

Lung and trachea cancer analyses were consistently deviating towards excess risk in females. Lung cancer risk is increased in the offspring of lung cancer patients regardless of the affected parent’s sex, with offspring cancer male–female ratios skewing towards an excess in females^11^. Young never-smoker women have an abundance of lung adenocarcinoma^27^, and molecular studies support that the phenotype is distinct from what is observed in smokers, and likely contributed to by cancer syndromes and other predisposing genetic factors^28^. Tobacco smoking is known to increase the risk of lung cancer, and it has been a wide-spread habit in Finnish males aged 15–64 with roughly 35% having been daily smokers in the 1970s and 23% in 2010^29^. The incidence patterns of male lung cancer may thus be fundamentally different from other combinations in our analysis. A similar but less pronounced effect may also be present in females due to passive smoking^30^.

The cluster analysis shows three clusters of male thyroid cancer with high SCIRs and a very high posterior probability of higher risk coefficient. A combination of a low background rate and an elevated familial risk in males, inverting the sex ratio when familial observations are compared to the sex ratio in background incidence, has previously been described in thyroid adenocarcinoma^11^. A similar effect may cause our cluster observations, with the exception that the morphological spectrum we observed was mixed.

Of all the clusters found in our data, the stomach cancer cluster with six patients was the most deviated from its respective group (median SCIR 8.14 standard deviations from male stomach cancer SCIR median as indicated by normalised SCIR). Stomach adenocarcinomas and carcinoid tumours are observed in the tumour spectrums of multiple hereditary cancer syndromes^31,32^, but there seems to be no overlap in strong genetic predisposition to the two tumour types.

## Conclusions

Our results imply that familial aggregation of cancers shows no sex-specific preference. However, the atypical sex-specific aggregation of stomach cancer, colon cancer, thyroid cancer and chronic lymphocytic leukaemia in certain families is difficult to fully explain with present knowledge of possible causes, and could yield useful knowledge if explored further.

## Supplementary Information

Supplementary Information 1.

Supplementary Information 2.

## Data Availability

The data underlying this study cannot be shared due to permissions that restricts our ability to share the research data on ethical and legal grounds. Similar permission to use administrative health data from various register keepers can be applied from Findata, the Social and Health Data Permit Authority. Requests to access these data can be submitted to Findata: https://findata.fi/en/.

## Abbreviations

ALL: Acute lymphoblastic leukaemia/lymphoma
AML: Acute myeloid leukaemia
Biliary (in Fig. 1): Gallbladder and bile ducts
CLL: Chronic lymphocytic leukaemia
CML: Chronic myeloid leukaemia
CNS: Brain, meninges, and central nervous system
FAP: Familial adenomatous polyposis
FCR: Finnish Cancer Registry
HL: Hodgkin lymphoma
Larynx (in Fig. 1): Larynx and epiglottis
Lung (in Fig. 1): Lung and trachea
Melanoma (in Fig. 1): Melanoma of the skin
MN: Municipality-name
MNS: Municipality-name-sex
Myeloma (in Fig. 1): Myeloma and other plasma cell tumours
Nasal (in Fig. 1): Nose and sinuses
PIS: Population Information System
Rectal (in Fig. 1): Rectum and rectosigmoid
SCIR: Standardised cumulative incidence ratio
SG: Salivary glands
SI: Small intestine
TG: Thyroid gland
Urinary (in Fig. 1): Bladder and urinary tract
