# Supplementary material for: Sex-specific familial aggregation of cancers in Finland
Source: Sci Rep. 2022 Sep 6;12:15126. doi: 10.1038/s41598-022-19039-1 (PMC9448814; doi:10.1038/s41598-022-19039-1)
Supplement: Supplementary file 1 — Supplementary Information 1. [file 41598_2022_19039_MOESM1_ESM.pdf]

Sex-specific familial aggregation of cancers in Finland

Statistical modeling and simulation details

Lauri J. Sipilä [a,b], Karri Seppä [c], Mervi Aavikko [a,b,d], Janne Ravantti [a,b,e], Sanna Heikkinen [c], Lauri A. Aaltonen [a,b], Janne Pitkäniemi [c,f,g,*]

Affiliations

[a] Department of Medical and Clinical Genetics, University of Helsinki, Biomedicum Helsinki, PO Box 63 (Haartmaninkatu 8), FI-00014, Helsinki, Finland

[b] Applied Tumor Genomics, Research Programs Unit, University of Helsinki, Biomedicum Helsinki, PO Box 63 (Haartmaninkatu 8), FI-00014, Helsinki, Finland

[c] Finnish Cancer Registry, Unioninkatu 22, 00130, Helsinki, Finland

[d] Institute for Molecular Medicine Finland (FIMM), HiLIFE, University of Helsinki, Helsinki, Finland

[e] Molecular and Integrative Biosciences Research Programme, Faculty of Biological and Environmental Sciences, University of Helsinki, FI-00014 Finland;

[f] Faculty of Social Sciences (Health Sciences), Tampere University, Tampere, Finland.

[g] Faculty of Medicine, University of Helsinki, Helsinki, Finland.

*Correspondence

Janne Pitkäniemi, Ph.D.
Professor of Cancer Epidemiology
Director of Statistics, Finnish Cancer Registry Institute for Statistical and Epidemiological Cancer Research
Finnish Cancer Registry, Unioninkatu 22, 00130, Helsinki, Finland
e-mail: janne.pitkaniemi@cancer.fi
Tel: +358 50 372 3335

Data can be presented using the observed number o_k and the expected number e_k of cancer cases in each combination k of family name and municipality. The expected number was calculated as the sum over calendar period specific expected numbers, i.e., $e_k = \Sigma_b\, C_b^* \times n_{bk}$ where cumulative cancer incidence proportion C_b^* in people born in Finland in period b is multiplied by the number n_{bk} of persons born in combination k in period b. A Poisson regression model was used to model the variation in the standardized cumulative incidence ratio $SCIR = \lambda = E(o/e)$ between combinations such that o_k~Poisson($\lambda_k e_k$) where

$$\log(E[o_k]) = \log(e_k) + \alpha + (1 - I_k)\beta_{k1} + I_k\beta_{k2}.$$

Random variable I_k follows the Bernoulli distribution and assigns combination k to low or high-risk category with probability $1 - \pi$ and π, respectively. Variation in cumulative cancer risk between combinations is modelled by effects β_{k1} and β_{k2}:

$$\beta_{k1} = \min(\omega_{k1}, \omega_{k2}) \text{ and } \beta_{k2} = \max(\omega_{k1}, \omega_{k2}) \text{ for all } k$$

where effect β_{k1} in the low-risk and β_{k2} in the high-risk group are based on iid random effects ω_{kj} that follow a normal distribution with mean zero and standard deviation σ.

The regression model was fit separately on the data of each site and sex using Markov chain Monte Carlo simulation. Vague N$(0, 100^2)$ priors were assumed for the fixed baseline effect α. For the probability π of the higher risk effect we assumed Unif$(0, 1)$ prior and for the hierarchical standard deviation σ Unif$(0, 2)$ prior. We ran a series of 125,000 iterations, of which the first 25,000 iterations were discarded as burn-in and every 20th of the remaining 100,000 iterations was stored and the posterior inferences were based on the sample of 5,000 values. In addition, two other series of 125,000 iterations were run in order to assess the convergence. Potential scale reduction factors [1, 2] were calculated to assess the convergence of the Markov chain. The simulations were carried out using JAGS version 4.3.0 [3] and R package rjags version 4-6. R package coda version 0.19-2 [4] was used for the convergence diagnostic.

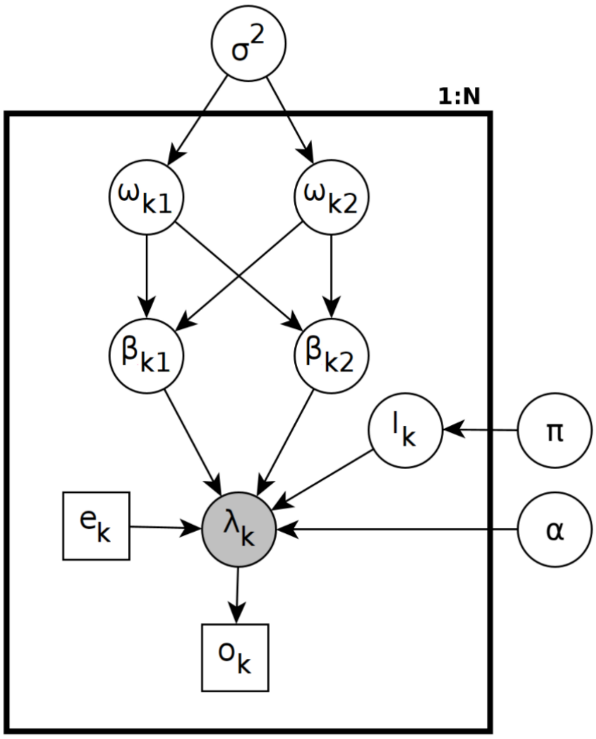

Fig. S1 A directed acyclic graph of the simulations. N is the total number of MNS combinations for a site-and-sex grouping. Rectangular nodes represent observed values and circular nodes unobserved values.

References

1. Gelman A, Rubin DB. Inference from Iterative Simulation Using Multiple Sequences. Stat Sci. 1992;7:457–72.

2. Brooks SP, Gelman A. General Methods for Monitoring Convergence of Iterative Simulations. J Comput Graph Stat 1998;7:434.

3. Plummer M, Others. JAGS: A program for analysis of Bayesian graphical models using Gibbs sampling. In: Proceedings of the 3rd international workshop on distributed statistical computing. Vienna, Austria. 2003;1–10.

4. Plummer M, Best N, Cowles K, Vines K. CODA: Convergence Diagnosis and Output Analysis for MCMC. R News 2006;6:7–11.
